# Multiple skeletal muscle metastases from poorly differentiated gastric adenocarcinoma

**DOI:** 10.1186/s40792-015-0108-3

**Published:** 2015-10-15

**Authors:** Yuki Koga, Yoshifumi Baba, Kazuto Harada, Keisuke Kosumi, Hironobu Shigaki, Junji Kurashige, Takatsugu Ishimoto, Masaaki Iwatsuki, Yuji Miyamoto, Yasuo Sakamoto, Naoya Yoshida, Hideo Baba

**Affiliations:** Department of Gastroenterological Surgery, Graduate School of Medical Science, Kumamoto University, 1-1-1 Honjo, Kumamoto, 860-8556 Japan

**Keywords:** Gastric carcinoma, Skeletal muscle tumor, Metastasis

## Abstract

We report here a rare case of gastric carcinoma with multiple intramuscular metastases. A 71-year-old man presented with rapidly evolving swelling of his left thigh and severe pain. Three years earlier, he had undergone neoadjuvant chemotherapy followed by gastrectomy for advanced gastric cancer. A computed tomography scan showed unusual swellings in multiple skeletal muscles with no vessel or bone invasion. Importantly, the affected muscles did not contain distinct masses but were diffusely enlarged. Pathological examination of an open muscle biopsy showed a poorly differentiated adenocarcinoma, supporting a diagnosis of gastric cancer metastases in multiple skeletal muscles.

## Background

Gastric cancer is the fourth commonest human malignant disease and the second commonest cause of cancer-related death worldwide [1]. Complete resection is essential for cure. Nonetheless, even after curative resection, 50–60 % of patients relapse locally or with distant metastases. Gastric cancer has four broad patterns of recurrence: local recurrence either in the gastric bed or regional lymph nodes, peritoneal dissemination, liver metastasis, and distant metastasis. A Japanese study of 939 patients who had undergone surgery for gastric cancer found that recurrence was local in 22 % of cases, peritoneal in 43 %, hepatic in 33 %, and distant in 21 %; 25 % of patients had recurrences in multiple sites [2]. Reported cases of skeletal muscle metastases from gastric carcinoma are extremely rare. We report here a case of a patient with swelling in the thigh that was diagnosed as skeletal muscle metastases from gastric carcinoma.

## Case presentation

**Fig. 1:**
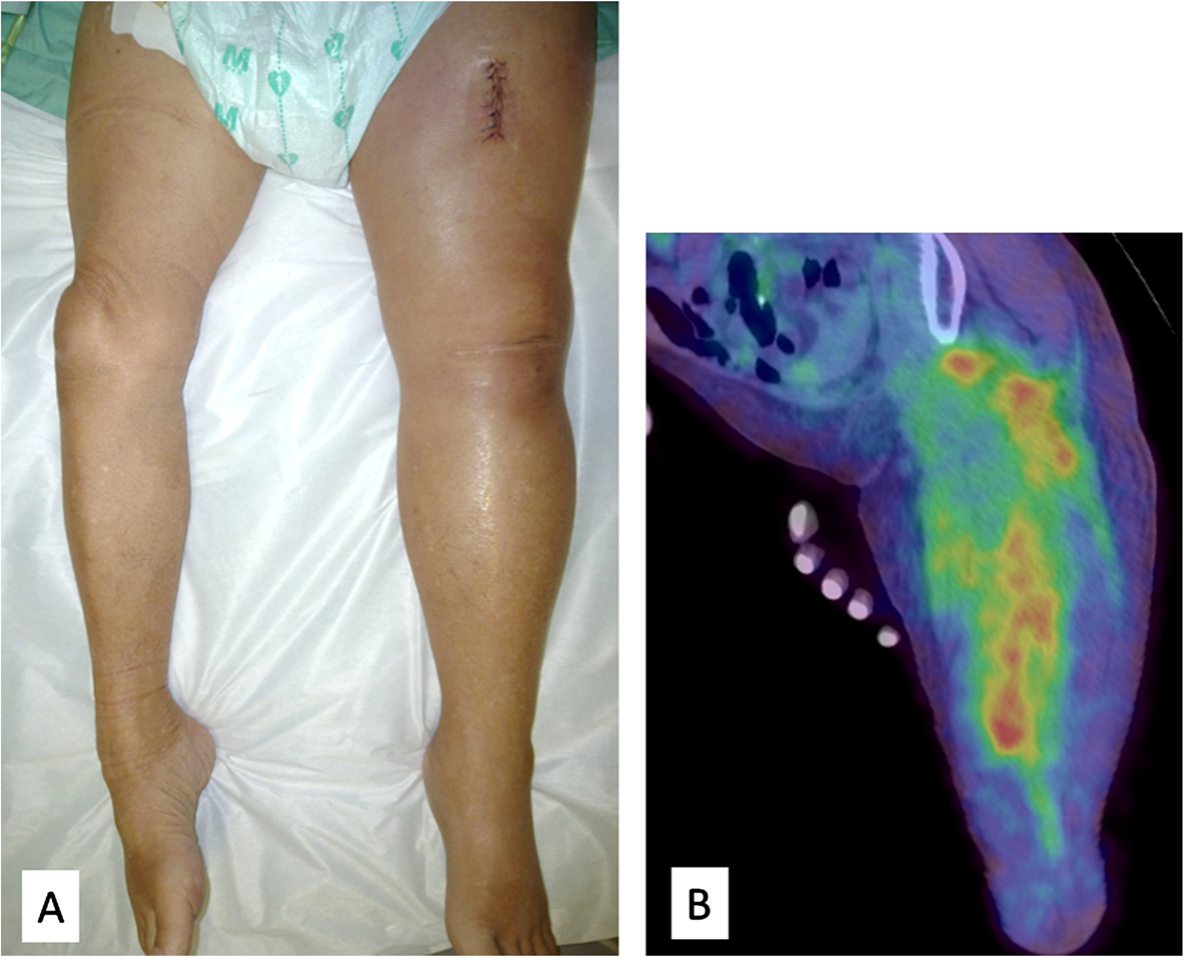
Photograph and positron emission tomography-computed tomography (PET-CT) image of the left femur. **a** Swelling of the left femur. An open muscle biopsy was performed. **b** PET-CT scan image showing increased metabolic activity in multiple skeletal muscles (latissimus dorsi, transverse, iliac, iliopsoas, and femoral)

**Fig. 2:**
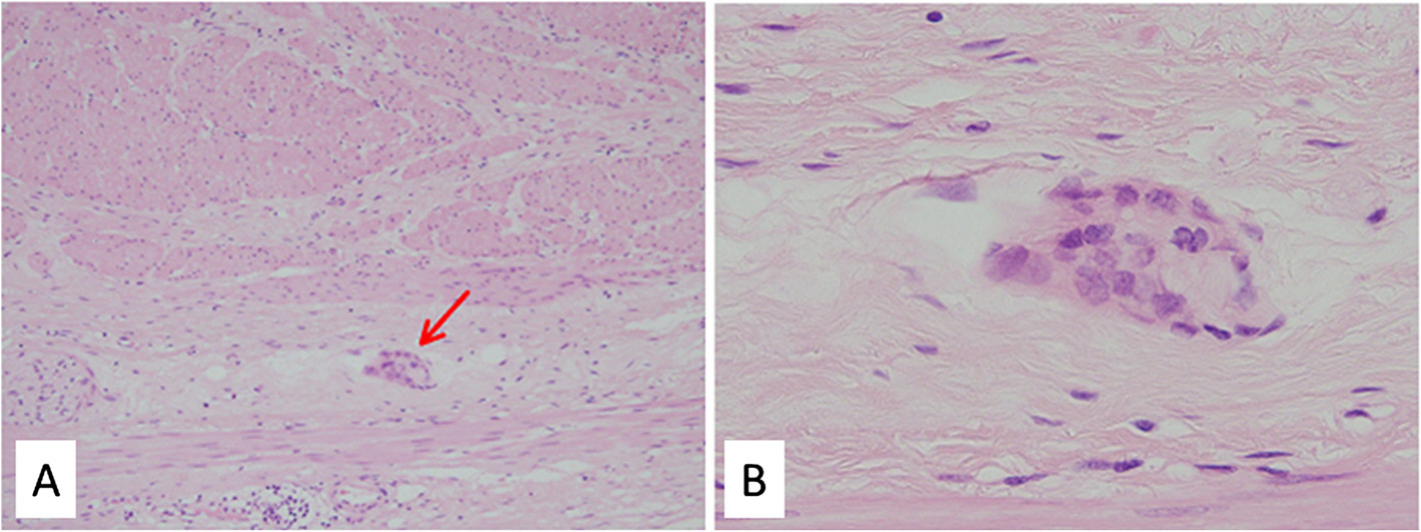
Microscopic appearance of the resected stomach. **a** Small clump of cancer cells in the muscularis propria. **b** Magnified image of adenocarcinoma cells in the muscularis propria

**Fig. 3:**
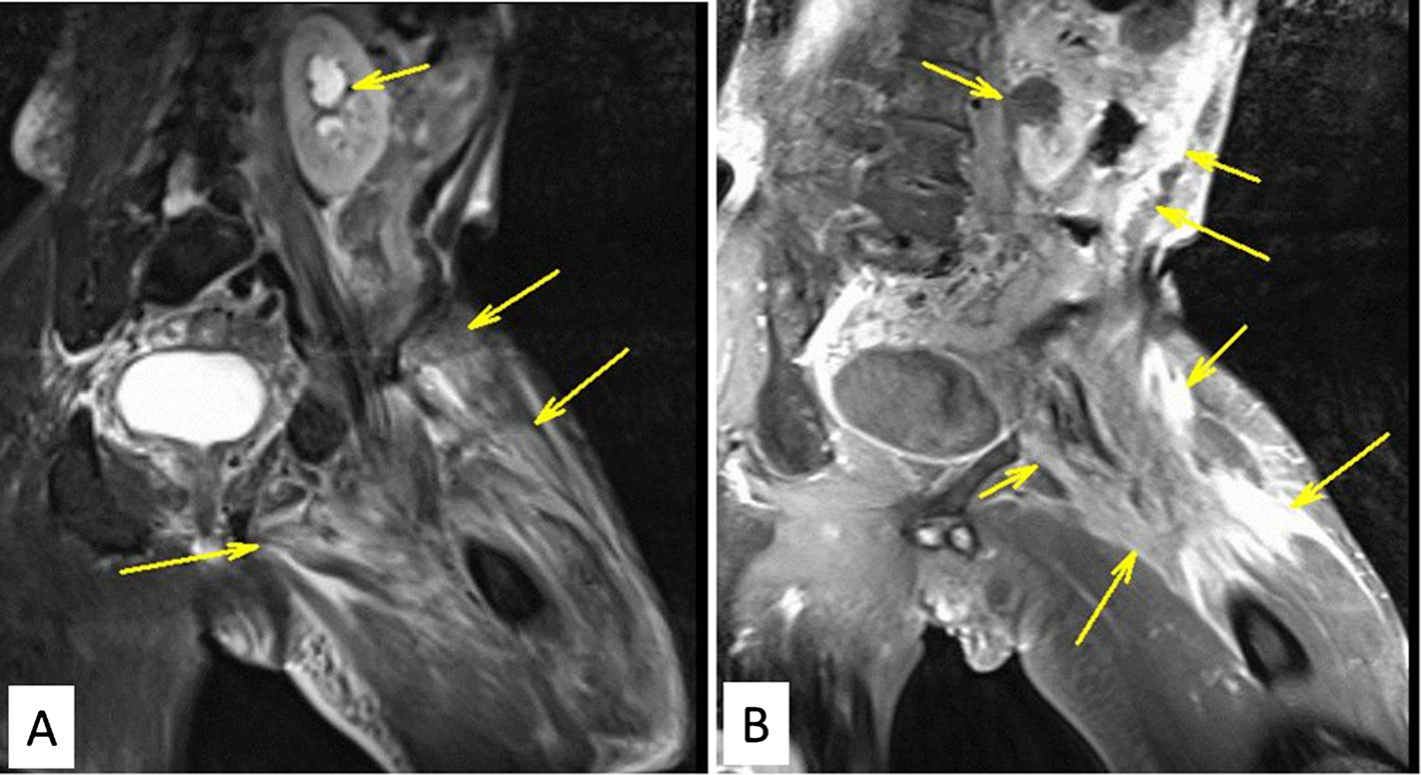
Magnetic resonance imaging (MRI) T1- and T2-weighted images. **a** MRI T2-weighted image showing the intramuscular mass has isosignal intensity. **b** MRI T1-weighted images showing heterogeneous intramuscular masses

**Fig. 4:**
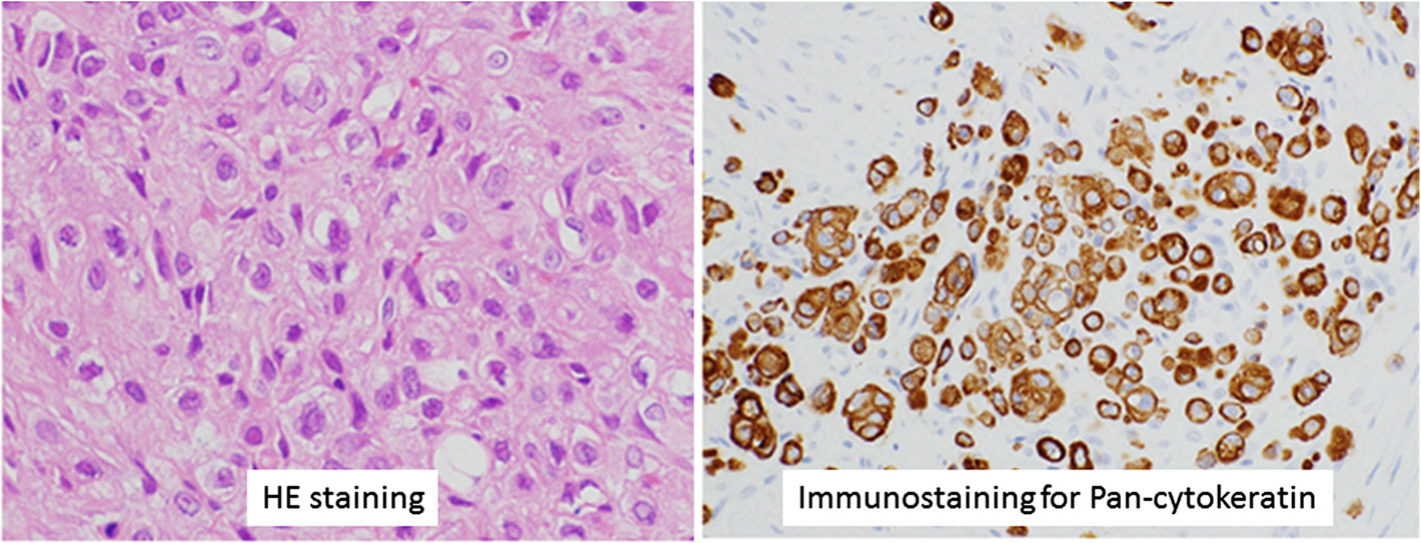
Microscopic appearance of the metastatic adenocarcinoma in the femoral muscle. (*Left*) H&E staining shows poorly differentiated adenocarcinoma. (*Right*) Positive immunohistochemistry for pan-cytokeratin

A 71-year-old man presented with a rapidly evolving swelling of his left thigh and severe pain. The circumference of his left thigh was 56.7 cm and his right thigh was 36.8 cm (Fig. 1). Three years earlier, he had undergone preoperative chemotherapy (cisplatin + S1) and total gastrectomy for advanced gastric cancer. The tumor was completely resected, the pathological diagnosis being adenocarcinoma, and small clump of cancer cells having been found in the muscularis propria (pathological stage T2N0M0 (AJCC classification), pathological effect grade 2) (Fig. 2). Immunohistochemistry showed that the cancer cells were positive for HER2. The patient received no postoperative chemotherapy. At follow-up, 2 years after surgery, concentrations of the tumor markers carcinoembryonic antigen (CEA) and carbohydrate antigen 19-9 (CA19-9) had increased. A computed tomography (CT) scan showed unusual swellings in multiple skeletal muscles (latissimus dorsi, transverse abdominal, iliac, iliopsoas, and femoral) with no vessel or bone invasion. Importantly, they did not contain distinct masses but were diffusely enlarged. A positron emission tomography (PET)-CT scan showed increased metabolic activity in these muscles. Magnetic resonance imaging (MRI) T1-weighted images showed heterogeneous intramuscular masses, whereas MRI T2-weighted images showed isosignal intensity. On MRI gadolinium-diethylene triamine pentaacetic acid (DTPA)-enhanced images, the intramuscular masses were enhanced with associated extensive peritumoral enhancement and central necrosis (Fig. 3). Our differential diagnosis included myositis, tubercular muscle abscess, primary soft-tissue sarcoma, and metastatic carcinoma. Pathological examination of an open muscle biopsy showed poorly differentiated adenocarcinoma, supporting a diagnosis of gastric cancer metastases in multiple skeletal muscles (Fig. 4). PET-CT scan showed no recurrent lesion other than multiple skeletal muscles. Based on pathological report demonstrating that the primary tumor was HER2-positive, the patient was commenced on chemotherapy with trastuzumab. However, as it turned out afterwards, cancer cells in the recurrent tumor were negative for HER2. In spite of this treatment, the concentrations of tumor markers (CEA and CA19-9) increased further, and the swellings in multiple skeletal muscles progressively enlarged. The patient died of respiratory failure with rapid collection of pleural effusion 54 days after admission, 18 days after establishment of the diagnosis of gastric cancer metastases.

## Discussion

Skeletal muscle metastases are rare [3–5]; the reported incidence ranging from 0.16–0.03 % in clinical practice and 0.8 % in an autopsy study [6]. The most common malignancies that metastasize to skeletal muscle are lung cancer (25 %), gastrointestinal tumors (21 %), urological tumors (13 %), genital tumors (9.3 %), and breast cancer (8.2 %) [7]. Muscle metastases most commonly occur in the lower limbs [5].

Gastric cancer rarely metastasizes to skeletal muscle, and such metastases are generally associated with widespread metastatic disease and poor prognosis. Because of their rarity and particular clinical characteristics, these metastases are challenging to diagnose [8]. To our knowledge, 21 cases of intramuscular metastases from gastric carcinoma, including our case, have been reported in the literature since 1970. These reported cases contain 17 males and 4 females, and their mean age was 62 years (range, 47–89 years) (Table 1) [3, 6, 8–24]. In some patients, including ours, metastases developed in multiple skeletal muscles. Our case was unusual for gastric carcinoma in that multiple muscle metastases developed without synchronous metastases to the liver or lungs. We were unable to determine the mechanism(s) of the metastases to skeletal muscle in our patient. However, given that skeletal muscle is a vascular tissue, we speculate that the mechanism of the muscle metastasis may be hematogenous.

**Table 1:** Reported cases of intramuscular metastasis from gastric carcinoma

Author	Year	Age	Sex	Muscle site of metastasis	Other site of metastasis	Stage	Treatment	
Traves et al.	1979	52	M	Psoas m.	N/A	N/A	N/A	[20]
Obley et al.	1983	54	M	Paraspinal m.	N/A	N/A	N/A	[15]
Rosenbaum et al.	1984	54	M	Upper arm m, femoral m.	N/A	TXNXM1	CRT	[3]
Arnold et al.	1989	59	F	Extraocular m.	N/A	N/A	N/A	[10]
Porlie et al.	1990	65	M	Sartorius m., rectus femoris m.	No other metastasis	TXNXM1	Chemotherapy	[17]
Sudo et al.	1993	61	M	Trapezius m.	N/A	N/A	N/A	[18]
Van Gelderen	1993	47	F	Extraocular m.	N/A	N/A	N/A	[21]
Toillon et al.	1994	68	M	Gastrocnemius m.	LNs around the esophageal hiatus and the celiac artery	TXNXM1	Chemotherapy	[19]
Amano et al.	1996	57	M	Gastrocnemius m.	N/A	TXNXM1	Chemotherapy and excision	[9]
Narvaez et al.	1998	49	M	Psoas m.	N/A	N/A	N/A	[13]
Pestalozzi et al.	1998	72	F	Gastrocnemius m.	Mediastinal LNs	T4N1M0	Excision and CRT	[16]
Oba et al.	2001	70	M	Lumbar muscle	Brain, lung, liver, bilateral adrenal glands, supraclavicular LN	TXNXM1	No treatment	[14]
Kondo et al.	2002	64	F	Gluteus maximus m., adductor magnus m.	Abdominal wall	T4N0M0	Excision	[12]
Touheti et al.	2004	48	M	Buttock	N/A	N/A	Excision	[23]
Touheti et al.	2004	89	M	Shoulder	N/A	N/A	Excision	[23]
Beşe NS et al.	2006	67	M	Posterior right paralumbar m. and posterior left paradorsal m.	Perigastric and lumboaortic LNs	Stage IV	Radiotherapy (palliative)	[24]
D. Tougeron et al.	2009	71	M	Deltoid muscle	No other metastasis	T4N1M0	Excision and CRT	[6]
Pinelopi V et al.	2012	-	M	Muscles of the left thigh	No other metastasis	T3N1M0	Excision	[11]
Ilaria Pergolini et al.	2014	47	M	Gluteus m.	Lumboaortic LN	TXNXM1	Chemotherapy	[8]
Lourenco et al.	2014	68	M	Right thigh	No other metastasis	TXNXM1	Chemotherapy	[22]
Our case	2015	71	M	Multiple (dorsal m., transverse abdominal m., iliac m., iliopsoas m., femoral m.)	No other metastasis	T2N0M0	Chemotherapy and excision	

*Abbreviations*: *CRT* chemoradiotherapy, *LN* lymph node, *N/A* not available

Most skeletal muscle metastases are detected on CT scan because such scans are routinely performed for oncologic staging. Unenhanced CT scans show intramuscular metastatic masses as isodense lesions compared with the surrounding muscle tissue. MRI is considered superior to CT scanning for detecting and characterizing muscle abnormalities [4, 23, 25]. Metastases in muscle frequently have isointense signals and ill-defined margins on T1-weighted MRI, whereas T2-weighted images generally show heterogeneous signal intensity with well-defined margins together with peritumoral edema [6, 25]. Tuoheti et al. showed that extensive peritumoral enhancement associated with central necrosis are characteristic features of skeletal muscle metastases on gadolinium-DTPA-enhanced MRI, these characteristics being found in 92 % of their cases [23]. Other features of intramuscular metastases include muscle enlargement, reticulated texture, and intratumoral patterns such as hemorrhage and central necrosis [12]. The radiological findings in our case were similar to those previously reported.

Therapeutic options for muscle metastases include radiotherapy, chemotherapy, and surgical excision [4, 23]. Radiotherapy can relieve the pain and decrease the size of such lesions [3, 6, 23, 24]. In carefully selected patients, surgical excision may help to relieve pain and prolong survival time. Chemotherapy, the only systemic option, is indicated when—as is usually the case—there is advanced disease with multiple metastatic sites [4, 23]. In our case, chemotherapy with a molecularly targeted agent (trastuzumab) did not prolong our patient’s life.

## Conclusions

The diagnosis of skeletal muscle metastasis should be considered in the differential diagnosis of any painful soft tissue mass because there are no clinical or radiographic characteristics that distinguish metastatic carcinoma in muscle from soft tissue sarcomas. However, extensive peritumoral enhancement on MRI should suggest skeletal muscle metastases.

## Consent

Written informed consent was obtained from the next of kin of the patient for publication of this case report and any accompanying images.
